# A Novel Method for Detecting Lanthanum Phosphate Deposition in the Gastroduodenal Mucosa Using Fluorescence Microscopy

**DOI:** 10.7759/cureus.30729

**Published:** 2022-10-26

**Authors:** Masaya Iwamuro, Haruo Urata, Satoshi Iwasa, Takehiro Tanaka, Yoshiro Kawahara, Horoyuki Okada

**Affiliations:** 1 Department of Gastroenterology and Hepatology, Okayama University Hospital, Okayama, JPN; 2 Central Research Laboratory, Okayama University Medical School, Okayama, JPN; 3 Department of Pathology, Okayama University Hospital, Okayama, JPN; 4 Department of Practical Gastrointestinal Endoscopy, Okayama University Hospital, Okayama, JPN; 5 Department of Gastroenterology and Hepatology, Okayama University Graduate School of Medicine, Dentistry, and Pharmaceutical Sciences, Okayama, JPN

**Keywords:** fluorescence microscopy, esophagogastroduodenoscopy, scanning electron microscopy analysis, lanthanum carbonate, hyperphosphatemia

## Abstract

Diagnostic utility of fluorescence microscopy for lanthanum phosphate deposition in the gastrointestinal mucosa has not been reported previously. In this study, we comparatively assessed the light, electron, and fluorescence microscopy features of gastroduodenal lanthanum phosphate deposition in 10 patients with deposits in the stomach and 5 patients with deposits in the duodenum. During light microscopy, lanthanum deposits were observed as dark-brown, needle-shaped, or crystalloid structures and pale red amorphous materials. During electron microscopy, the deposited material appeared as bright aggregates. Fluorescence microscopy also revealed lanthanum deposits as bright areas under green, red, and blue filters. The deposits were more easily recognizable on electron and fluorescence microscopy than on light microscopy. Furthermore, during fluorescence microscopy, the green filter provided the most clear visualization of lanthanum phosphate. In conclusion, fluorescence microscopy with a green filter is useful in determining the degree and extent of lanthanum deposition in the gastroduodenal mucosa.

## Introduction

Lanthanum carbonate is a calcium-free agent used to treat hyperphosphatemia in patients with chronic kidney disease [1]. It lowers the serum phosphorus levels by binding any phosphate that may be present in the ingested food. Although lanthanum phosphate is hardly absorbed via the intestinal mucosa, an increasing body of evidence suggests that lanthanum phosphate deposits are present in biopsy specimens collected from the gastroduodenal mucosa of patients who consume lanthanum carbonate [2-9]. Lanthanum can be found in a number of food items such as garden tomatoes, dill, broccoli, and almonds. However, the amount of lanthanum is at most 0.3 μg per 100 g of food, which is negligible compared with medication doses of 750 to 2,250 mg per day [10]. Even though health problems secondary to lanthanum phosphate deposition have not yet been reported, the long-term (>10 years) safety of lanthanum carbonate intake has not yet been elucidated, as its deposition in the gastroduodenal mucosa was first reported in 2015 [11]. Thus, accurate pathological diagnosis and monitoring the patients’ clinical course are essential to elucidate the pathological significance of lanthanum phosphate deposition in the gastroduodenal mucosa.

During conventional pathological analysis, massive lanthanum phosphate deposits in biopsy specimens can be easily identified during light microscopy with hematoxylin and eosin staining; however, trace amounts of lanthanum or partially deposited lanthanum may be overlooked. Recently, we observed that lanthanum phosphate deposition in the gastroduodenal mucosa clearly fluoresces during fluorescent microscopy. Therefore, in this study, we explored the diagnostic utility of fluorescence microscopy for gastroduodenal lanthanum phosphate deposits. We also investigated which excitation filter is the most appropriate for detecting these deposits in biopsy specimens.

## Materials and methods

A database search of endoscopic examination reports at the Department of Endoscopy, Okayama University Hospital, revealed 25 patients diagnosed with pathologically proven lanthanum phosphate deposition in the gastroduodenal mucosa between January 2013 and November 2017. All patients were undergoing dialysis and were taking lanthanum carbonate to treat or prevent hyperphosphatemia. Endoscopy was performed as part of the standard care for neoplasia screening, mostly on a yearly basis. In all patients, the presence of lanthanum and phosphate in the specimen was confirmed using scanning electron microscopy (SEM) and energy dispersive X-ray spectroscopic analyses. In this study, we enrolled 10 and 5 randomly selected patients with lanthanum phosphate deposition in the stomach and the duodenum, respectively, and comparatively assessed the light, electron, and fluorescence microscopy images between them.

SEM observation was performed as described in our previous studies [3,8,12]. Briefly, a paraffin-embedded section was deparaffinized with xylene (10 min, twice) and subsequently washed with a serial dilution of ethanol (100% for 5 minutes, thrice; 80% for 5 minutes; and 50% for 5 minutes). The surface of the sample was coated with osmium for 10 seconds (HPC-1S-type osmium coater; Shinku Device Co., Ibaraki, Japan) and SEM images were captured using the S4800 electron microscope (Hitachi, Tokyo, Japan).

For fluorescence microscopy, we used paraffin-embedded, unstained sections without deparaffinization and captured images using the FSX100 microscope (Olympus Co., Tokyo, Japan). We evaluated the visual recognizability of lanthanum phosphate on photographs using three channels: blue (bandpass filter: 360-370 nm, barrier filter: 420-460 nm, and dichroic mirror: 400 nm), green (bandpass filter: 460-495 nm, barrier filter: 510-550 nm, and dichroic mirror: 505 nm), and red (bandpass filter: 530-550 nm, interference barrier filter: 575 nm, and dichroic mirror: 570 nm). To investigate the fluorescence of lanthanum phosphate, we placed lanthanum (III) phosphate hydrate (Sigma-Aldrich Co., St. Louis, MO) on a glass slide and observed the same under the fluorescence microscope.

This retrospective study was approved by the Ethics Committee of Okayama University Hospital and adhered to the principles of the Declaration of Helsinki.

## Results

**Figure 1:**
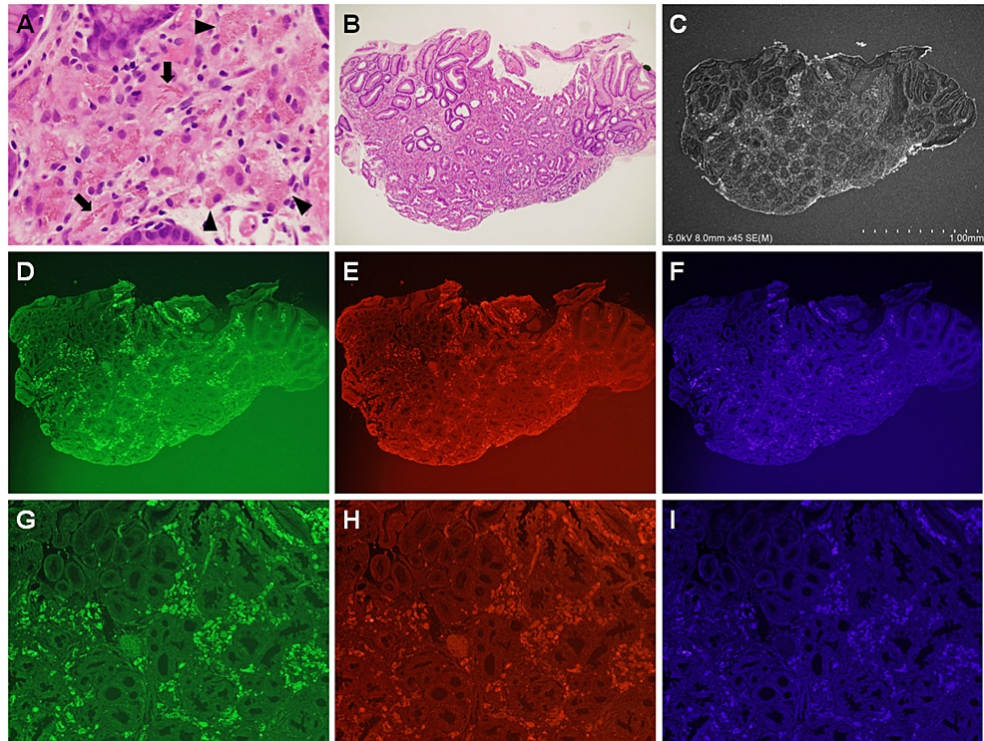
Microscopy images of lanthanum phosphate deposition in the stomach (case 1). Lanthanum phosphate deposits in hematoxylin and eosin-stained tissues are observed as dark-brown, needle-shaped, or crystalloid structures (A: ×40, arrows), or as pale red, amorphous materials (A, arrowheads). The deposited area is slightly harder to identify on a lower magnification image (B: ×4.2). During scanning electron microscopy, the deposited material appears as bright aggregates (C). Fluorescence microscopy also reveals lanthanum phosphate deposits as bright areas (D–F: ×4.2, G–I: ×20).

Fluorescence microscopy revealed lanthanum phosphate deposits as bright areas in all patients with gastric (N = 10, 100%) and duodenal lanthanum deposition (N = 5, 100%). Representative images of light, scanning electron, and fluorescence microscopic fields are shown in Figure 1. Visual recognizability of lanthanum phosphate was greater on electron (Figure 1C) and fluorescence (Figures 1D-1F) microscopy than on light microscopy (Figures 1A, 1B). Lanthanum phosphate deposits in hematoxylin and eosin-stained tissues (Figures 1A, 1B) were observed as either dark-brown, needle-shaped, or crystalloid structures (Figure 1A, arrows), or as pale red amorphous materials (Figure 1A, arrowheads). On SEM, the deposits appeared as bright aggregates (Figure 1C). Fluorescence microscopy also revealed lanthanum phosphate deposition as bright areas under green (Figure 1D), red (Figure 1E), and blue (Figure 1F) filters. The boundaries of lanthanum phosphate deposits were clearly visible in images of higher magnification (Figures 1G-1I). Among the three filters, visual recognizability was the highest under the green filter (Figures 1D, 1G).

**Figure 2:**
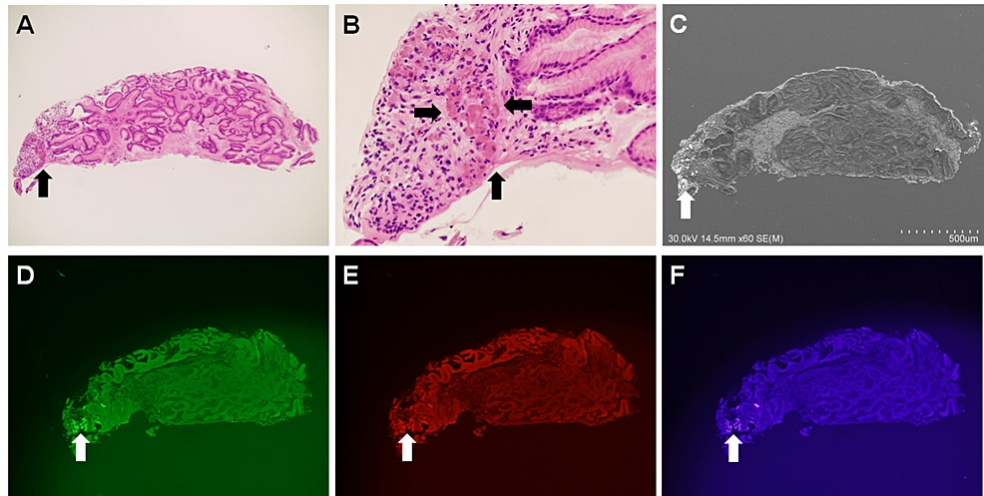
Microscopy images of lanthanum phosphate deposition in the stomach (case 2). Lanthanum phosphate is deposited in a limited area (A: ×4.2, B: ×20, hematoxylin and eosin stain, arrows). The deposited lanthanum phosphate is easily identifiable on scanning electron (C, arrow) and fluorescence microscopy images (D–F: ×4.2, arrows).

Figure 2 shows microscopy images from another gastric mucosal specimen with lanthanum phosphate deposition. In this specimen, lanthanum phosphate deposited in a limited area (Figures 2A, 2B) was easily identifiable on SEM (Figure 2C) and fluorescence microscopy (Figures 2D-2F) images.

**Figure 3:**
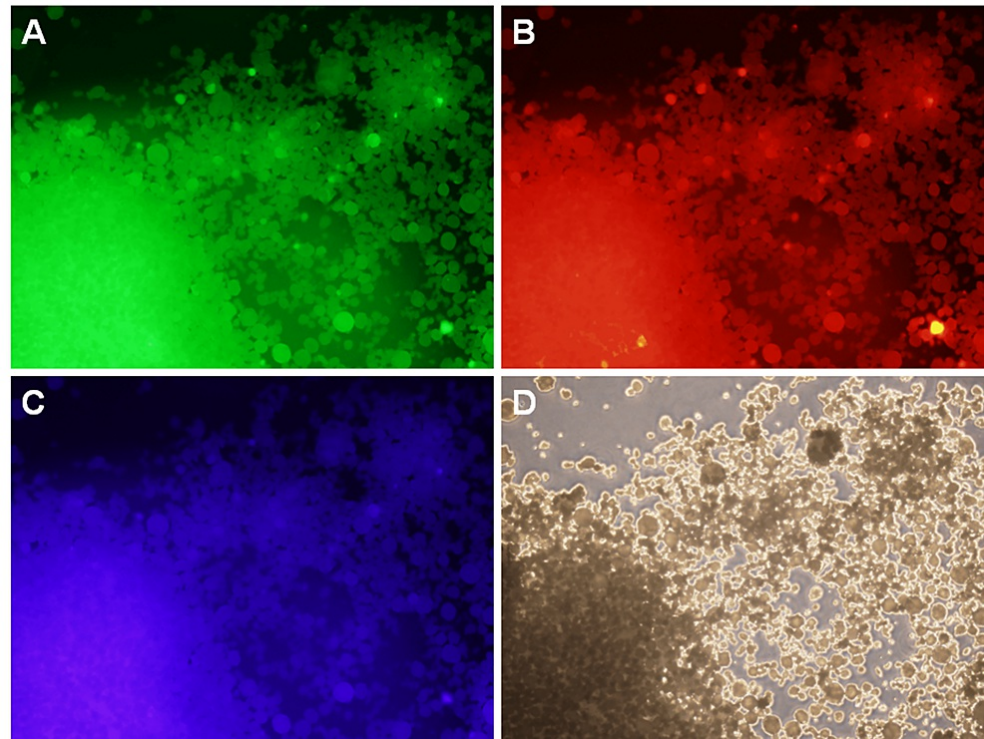
Fluorescence microscopy images of lanthanum (III) phosphate hydrate. Fluorescent signals are observed through the green (A), red (B), and blue (C) filters. These signals correspond to the lanthanum (III) phosphate hydrate powder (D: phase contrast microscopy image).

Observation of lanthanum (III) phosphate hydrate under fluorescence microscopy with green (Figure 3A), red (Figure 3B), and blue (Figure 3C) filters revealed fluorescent granules that corresponded to the phase-contrast microscopy image of lanthanum (III) phosphate hydrate powder (Figure 3D).

## Discussion

This study is the first to show that areas of lanthanum phosphate deposition are easily identifiable under fluorescence microscopy. As mentioned above, when observed under hematoxylin and eosin staining, the pathological features of lanthanum phosphate deposits vary from dark-brown, needle-shaped, or crystalloid, to pale red and amorphous. As there is little difference in the color between the latter form of lanthanum and the mucosal parenchyma, i.e., the background, it is often difficult to determine the extent of deposition, particularly in light microscopic images of lower magnification (Figure 1B). Moreover, the deposits may be overlooked when the amount is trace or the deposited area is limited (Figure 2A). As shown in the present study, fluorescence microscopy offers an advantage over light microscopy in terms of a clear visualization of lanthanum phosphate.

While the mechanisms behind the strong fluorescent signal of deposited lanthanum phosphate are unknown, lanthanum (III) phosphate hydrate appeared bright on fluorescence microscopy (Figure 3), suggesting autofluorescence. Although it was a subjective evaluation, fluorescent images captured under the green filter provided more favorable visibility properties than those captured under the red or blue filters. Consequently, we would like to propose that biopsied specimens be observed under fluorescence microscopy using a green filter to detect lanthanum phosphate deposition in patients who are taking lanthanum carbonate.

We have previously reported the usefulness of SEM in the detection of gastrointestinal lanthanum phosphate deposits [3,8]. Under SEM, the deposited material is visible as a bright area, which enables an easier identification (Figures 1C, 2C). However, deparaffinization and surface-coating with a thin conductive layer (such as osmium) are prerequisites for sample preparation (Table 1). Moreover, electron microscopes are not readily accessible in most pathology departments. Conversely, sample preparation is not required in fluorescence microscopy; one only has to observe the unstained sections without deparaffinization. Thus, although electron microscopy is advantageous in proving the presence of lanthanum and phosphate elements using energy-dispersive X-ray spectroscopic analyses, fluorescence microscopy is more convenient for the detection of the same deposited in the gastrointestinal tract.

**Table 1:** Methods to identify lanthanum deposition in the gastrointestinal tract

Diagnostic techniques	Deposited lanthanum identified as	Sample processing of formalin-fixed, paraffin-embedded tissue sections
Light microscopy, hematoxylin and eosin staining	Dark-brown, needle-shaped, or crystalloid structures and pale red amorphous materials	Hematoxylin and eosin staining
Light microscopy, CD68 staining	CD68-positive macrophages (which correspond to phagocytes containing lanthanum)	CD68 staining
Scanning electron microscopy	Bright aggregates	Deparaffinization and surface coating
Energy dispersive X-ray spectroscopy	Wavelengths specific to lanthanum element	Deparaffinization and surface coating
Fluorescence microscopy	Bright areas	None

In patients with chronic kidney disease undergoing dialysis, long-term exposure to aluminum and magnesium salts leads to osteomalacia, encephalopathy, dementia, and microcytic anemia. Consequently, we believe that the accumulation of inorganic substances, such as lanthanum, in the human body should be monitored for a prolonged period, even though health problems secondary to lanthanum phosphate deposition have not yet been reported. In addition, increased inflammation and worsening of erosion in the stomach of patients during the course of gastric lanthanum deposition have been reported [13]. We speculate that lanthanum deposition may damage the gastric mucosa. Histological alterations in rat stomachs after administration of lanthanum carbonate have also been reported [14]. Glandular atrophy, stromal fibrosis, proliferation of mucous neck cells, intestinal metaplasia, squamous cell papilloma, erosion, and ulcers were detected, indicating a potential to induce abnormal cell proliferation or neoplastic lesions. Thus, surveillance of gastric mucosal damage and neoplastic lesions may be required in patients with gastric lanthanum deposition. We believe that the evaluation of endoscopically biopsied specimens from the gastroduodenal mucosa using fluorescence microscopy will enable precise determination of the presence and degree of lanthanum phosphate deposition, which will reveal the pathological significance of this disease entity in the future.

## Conclusions

We reported the usefulness of fluorescence microscopy for determining the degree and extent of lanthanum phosphate deposition and for the detection of material deposited in a limited area. We hope that this report will help pathologists to appropriately diagnose and evaluate patients with lanthanum phosphate deposition in the gastrointestinal mucosa.
